# Complete mitochondrial genome of *Mollitrichosiphum tenuicorpus* (Okajima, 1908) (Hemiptera: Aphididae: Greenideinae)

**DOI:** 10.1080/23802359.2020.1866462

**Published:** 2021-02-08

**Authors:** Cailing Li, Liyun Jiang, Gexia Qiao, Jing Chen

**Affiliations:** a Key Laboratory of Zoological Systematics and Evolution, Institute of Zoology, Chinese Academy of Sciences, Beijing, PR China; b College of Life Sciences, University of Chinese Academy of Sciences, Beijing, PR China

**Keywords:** Aphids, mitogenome, phylogeny, repeat region

## Abstract

Here we report the complete mitochondrial genome of the aphid species *Mollitrichosiphum tenuicorpus*. The *M. tenuicorpus* mitogenome is 15,727 bp in length and comprising 37 genes typically present in insect mitogenomes, a control region, and a unique repeat region. All protein-coding genes (PCGs) terminate with TAA or TAG except for *cox1*, which is terminated with T—. The secondary structure of *trnS (AGN)* loses the dihydrouridine (DHU) arm, but all the other transfer RNAs show the typical clover-leaf secondary structure. The repeat region between *trnE* and *trnF* is 458 bp, with a 217-bp repeat unit repeating 2.11 times. Phylogenetic analysis of the *M. tenuicorpus* mitogenome using the maximum-likelihood optimality criterion places it in a strongly supported sister position to *Eutrichosiphum pasaniae*. These data show that mitogenome sequences could be useful in resolving phylogenetic relationships of the Greenideinae.

The aphid species *Mollitrichosiphum tenuicorpus* (Okajima, 1908) (Aphididae: Greenideinae: Greenideini) is widely distributed in southeastern Asia. It is monoecious and holocyclic, mainly feeding on young shoots of the plants of *Castanopsis*, *Litsea*, and *Lithocarpus* (Blackman and Eastop 2020). To date, five complete mitochondrial genomes of Greenideinae species have been reported (Wang et al. 2014; Chen et al. 2019, 2020; Li et al. 2020; Liu et al. 2020). In this study, we sequenced and annotated the mitochondrial genome of *M. tenuicorpus*. The aphid specimens were collected on *Lithocarpus glaber* from Lishui City, Zhejiang, China (27.6909°N, 119.6352°E) and deposited in the National Zoological Museum of China, Institute of Zoology, Chinese Academy of Sciences, Beijing, China (NZMC no. 38234, Jing Chen, chenjing@ioz.ac.cn). The sequencing was performed on an Illumina platform. The mitogenome was assembled using SPAdes version 3.10.1 (Bankevich et al. 2012) and the annotation was conducted using MITOS version 2 WebServer (Bernt et al. 2013), followed by manual adjustments.

The mitochondrial genome of *M. tenuicorpus* is 15,727 bp long, with an A + T content of 84.5% (GenBank accession number MW123009). The mitogenome contains 13 protein-coding genes (PCGs), 22 transfer RNA genes (tRNAs), 2 ribosomal RNA genes (rRNAs), a control region, and a special non-coding repeat region. The gene order is identical to the inferred ancestral arrangement of insects (Clary and Wolstenholme 1985). All PCGs are initiated by ATN codons. Twelve PCGs are terminated with TAA or TAG codons, whereas *cox1* uses the incomplete T— as the stop codon. The tRNA genes range from 62 to 73 bp in length. All tRNAs except for *trnS* (*AGN*) display the typical clover-leaf secondary structure. The dihydrouridine (DHU) arm is lost in the secondary structure of *trnS (AGN)*. The *rrnL* and *rrnS* genes are located on the minority strand. The *rrnL* gene is 1271 bp long, with an A + T content of 85.7%. The *rrnS* gene is 769 bp in length, with an A + T content of 83.2%. The control region resides between *rrnS* and *trnI*. It is 680 bp long with an A + T content of 90.5%, including an AT-rich zone, a poly-thymidine stretch, and a stem-loop region. In the mitogenome of *M. tenuicorpus*, the repeat region located between *trnE* and *trnF* is 458 bp in length with an A + T content of 87.2%. Within this non-coding repeat region, a 217-bp repeat unit repeats 2.11 times. Up to now, this aphid-specific repeat region has been found in all reported mitogenomes of Greenideinae species except for *Cervaphis quercus* Takahashi, 1918.

We built a maximum-likelihood tree of aphids using the whole mitogenome sequences of *M. tenuicorpus* and 26 other aphid species. The phylogenetic analysis was performed using RAxML version 8.2.10 (Stamatakis 2014). All subfamilies represented by more than one aphid species were recovered as monophyletic except for Eriosomatinae. The monophyly of Greenideinae and Greenideini was well supported with high bootstrap values. The tribe Cervaphidini was sister to Schoutedeniini + Greenideini, which is consistent with a previous phylogenetic study of Greenideinae based on multiple nuclear and mitochondrial genes (Liu et al. 2015). Within the clade of Greenideini, *M. tenuicorpus* and *Eutrichosiphum pasaniae* formed a sister group with strong support (Figure 1).

**Figure 1.**
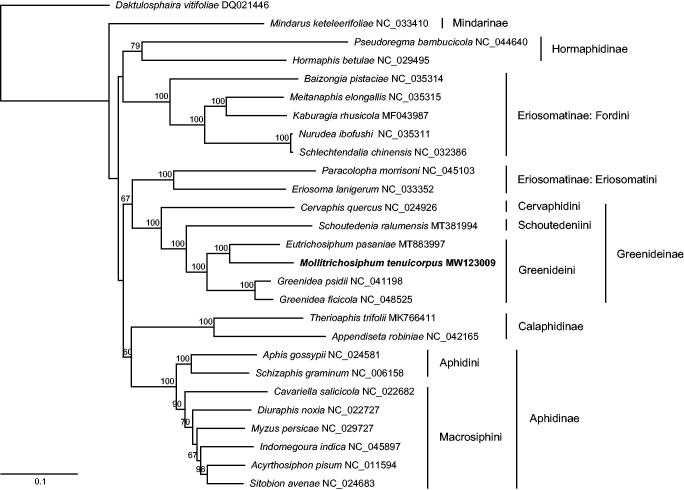
The maximum-likelihood tree inferred from the whole mitochondrial genomes of *M. tenuicorpus* and 26 other aphids. Bootstrap values (>50%) are shown above the branches.

## Data Availability

The genome sequence data that support the findings of this study are openly available in GenBank of NCBI at https://www.ncbi.nlm.nih.gov under the accession no. MW123009. The associated BioProject, SRA and Bio-Sample numbers are PRJNA681930, SRX9616531 and SAMN16974884, respectively.
